# DNA as a medium for hiding data

**DOI:** 10.1186/1471-2105-13-S12-A23

**Published:** 2012-07-31

**Authors:** Marc Beck, Roman Yampolskiy

**Affiliations:** 1 Department of Computer Engineering and Computer Science, University of Louisville, Louisville, KY 40292, USA

## Background

Steganography is the science of hiding information by transmitting secret messages through unsuspicious cover carriers in a way that makes the presence of any embedded messages undetectable. The term originates in the Greek language and means, "covered writing". While the goal of cryptography is to make a message unreadable, steganography aims at avoiding suspicion to the existence of a hidden message. A coding scheme is a set of rules that determines which symbol of the source alphabet is represented by which symbol in the target alphabet. Several different coding schemes have been developed within the past decade by different groups of researchers. The coding schemes differ in codon length, detectability, number of characters that can be encoded, and the number of steps involved in encoding a message. Recent advances in genetic engineering have allowed the insertion of artificial DNA strands into the living cells of organisms. Instead of expressing a message as a series of ones and zeros, it is represented in DNA code as a series of As, Cs, Gs, and Ts. A number of algorithms have been developed to encode a message in DNA characters and either disguise these messages as novel DNA sequences or encapsulate them within existing ones. In this work we describe methods to insert information into a DNA sequence for the purpose of data storage, watermarking, or communication of secret messages.

